# Changes to publication requirements made at the XVIII International Botanical Congress in Melbourne - what does e-publication mean for you?

**DOI:** 10.1186/1471-2148-11-250

**Published:** 2011-09-14

**Authors:** Sandra Knapp, John McNeill, Nicholas J Turland

**Affiliations:** 1 Department of Botany, The Natural History Museum, Cromwell Road, London SW7 5BD, UK; 2 Royal Botanic Garden, Edinburgh, 20A Inverleith Row, Edinburgh EH3 5LR, UK; 3 Missouri Botanical Garden, PO Box 299, St Louis, MO 63166-0299, USA

## Abstract

Changes to the *International Code of Botanical Nomenclature *are decided on every 6 years at Nomenclature Sections associated with International Botanical Congresses (IBC). The XVIII IBC was held in Melbourne, Australia; the Nomenclature Section met on 18-22 July 2011 and its decisions were accepted by the Congress at its plenary session on 30 July. Several important changes were made to the *Code *as a result of this meeting that will affect publication of new names. Two of these changes will come into effect on 1 January 2012, some months before the *Melbourne Code *is published. Electronic material published online in Portable Document Format (PDF) with an International Standard Serial Number (ISSN) or an International Standard Book Number (ISBN) will constitute effective publication, and the requirement for a Latin description or diagnosis for names of new taxa will be changed to a requirement for a description or diagnosis in either Latin or English. In addition, effective from 1 January 2013, new names of organisms treated as fungi must, in order to be validly published, include in the protologue (everything associated with a name at its valid publication) the citation of an identifier issued by a recognized repository (such as MycoBank). Draft text of the new articles dealing with electronic publication is provided and best practice is outlined.

To encourage dissemination of the changes made to the International Code of Nomenclature for algae, fungi, and plants, this article will be published in *BMC Evolutionary Biology, Botanical Journal of the Linnean Society, Brittonia, Cladistics, MycoKeys, Mycotaxon, New Phytologist, North American Fungi, Novon, Opuscula Philolichenum, PhytoKeys, Phytoneuron, Phytotaxa, Plant Diversity and Resources, Systematic Botany* and *Taxon*.

## Introduction

At the XVIII International Botanical Congress in Melbourne, Australia, in July 2011, two important changes were made to the *International Code of Botanical Nomenclature *(now the *International Code of Nomenclature for algae, fungi, and plants*) that will take effect from 1 January 2012. These changes will affect everyone who publishes names governed by this *Code*. As the *Melbourne Code *will not be published until approximately mid-2012, we felt it would be helpful to outline these changes, particularly those concerning effective publication in electronic media (in Articles 29, 30, and 31). For a concise report on all the changes to the *Code *accepted in Melbourne, see [1].

A draft wording of the revised Articles, Notes, and Recommendations on effective publication is provided to aid editors and publishers in establishing best practice for implementing this aspect of the *Code*. We also outline here what these changes do *not *mean, to guide those wishing to publish new names and typifications by electronic means. We urge readers to consult the report of the Special Committee on Electronic Publication accompanying the changes proposed before the Congress [2], wherein the reasoning for the changes now accepted into the *Code *is set out.

## Draft wording of revised Articles 29, 30, and 31 and Recommendations 29A, 30A, and 31A

Here we reproduce the wording of all of the relevant Articles, Notes, and Recommendations (omitting the Examples), with the changes highlighted in **bold**. The wording here is provisional, pending the meeting of the Editorial Committee in December 2011 to finalize the printed version of the *Melbourne Code*.

Article 29

*29.1*. Publication is effected, under this *Code*, by distribution of printed matter (through sale, exchange or gift) to the general public or at least to botanical institutions with libraries accessible to botanists generally. **Publication is also effected by electronic distribution of material in Portable Document Format (PDF; see also Art. 29.3 and Rec. 29A.1) in an online publication with an International Standard Serial Number (ISSN) or an International Standard Book Number (ISBN)**. Publication is not effected by communication of new names at a public meeting, by the placing of names in collections or gardens open to the public, by the issue of microfilm made from manuscripts, typescripts or other unpublished material, or by distribution electronically **other than as described above**.

***29.2*. For the purpose of this Article, "online" is defined as accessible electronically via the World Wide Web**.

***29.3*. Should Portable Document Format (PDF) be succeeded, a successor international standard format communicated by the General Committee (see Div. III) is acceptable**.

***29.4*. The content of a particular electronic publication must not be altered after it is first issued. Any such alterations are not themselves effectively published. Corrections or revisions must be issued separately to be effectively published**.

Recommendation 29A

[Existing Recommendation replaced by the following:]

***29A.1*. Publication electronically in Portable Document Format (PDF) should comply with the PDF/A archival standard (ISO 19005)**.

*29A.2*. Authors should preferably publish in publications that are archived, satisfying the following criteria as far as is practical (see also Rec. 29A.1):

*(a) *The material should be placed in multiple trusted online digital repositories, e.g. an ISO-certified repository;

*(b) *Digital repositories should be in more than one area of the world and preferably on different continents;

***(c) *Deposition of printed copies in libraries in more than one area of the world and preferably on different continents is also advisable**.

Article 30

***30.1*. Publication by distribution of electronic material does not constitute effective publication before 1 January 2012**.

***30.2*. An electronic publication is not effectively published if there is evidence associated with or within the publication that it is merely a preliminary version that was, or is to be, replaced by a version that the publisher considers final, in which case only that final version is effectively published**.

*30.3*. Publication by indelible autograph before 1 January 1953 is effective. Indelible autograph produced at a later date is not effectively published.

*30.4*. For the purpose of this Article, indelible autograph is handwritten material reproduced by some mechanical or graphic process (such as lithography, offset, or metallic etching).

*30.5*. Publication on or after 1 January 1953 in trade catalogues or non-scientific newspapers, and on or after 1 January 1973 in seed-exchange lists, does not constitute effective publication.

*30.6*. The distribution on or after 1 January 1953 of printed matter accompanying exsiccatae does not constitute effective publication.

*Note 1*. If the printed matter is also distributed independently of the exsiccata, it is effectively published.

*30.7*. Publication on or after 1 January 1953 of an independent non-serial work stated to be a thesis submitted to a university or other institute of education for the purpose of obtaining a degree is not effectively published unless it includes an explicit statement (referring to the requirements of the *Code *for effective publication) or other internal evidence that it is regarded as an effective publication by its author or publisher.

*Note 2*. The presence of an International Standard Book Number (ISBN) or a statement of the name of the printer, publisher, or distributor in the original printed version is regarded as internal evidence that the work was intended to be effectively published.

Recommendation 30A

***30A.1*. Preliminary and final versions of the same electronic publication should be clearly indicated as such when they are first issued**.

*30A.2*. It is strongly recommended that authors avoid publishing new names and descriptions or diagnoses of new taxa (nomenclatural novelties) in ephemeral printed matter of any kind, in particular printed matter that is multiplied in restricted and uncertain numbers, in which the permanence of the text may be limited, for which effective publication in terms of number of copies is not obvious, or that is unlikely to reach the general public. Authors should also avoid publishing new names and descriptions or diagnoses in popular periodicals, in abstracting journals, or on correction slips.

*30A.3*. To aid availability through time and place, authors publishing nomenclatural novelties should give preference to periodicals that regularly publish taxonomic articles. **Otherwise, a copy of a publication (whether published as printed or electronic matter) should be sent to an indexing centre appropriate to the taxonomic group, and publications that exist only as printed matter **should be deposited in at least ten, but preferably more, botanical or other generally accessible libraries throughout the world.

*30A.4*. Authors and editors are encouraged to mention nomenclatural novelties in the summary or abstract, or list them in an index in the publication.

Article 31

*31.1*. The date of effective publication is the date on which the printed **or electronic **matter became available as defined in Art. 29 and 30. In the absence of proof establishing some other date, the one appearing in the printed **or electronic **matter must be accepted as correct.

[Existing Note 1 replaced by the following:]

***31.2*. When a publication is issued in parallel electronic and printed versions, these must be treated as effectively published on the same date unless the dates of the versions are different according to Art. 31.1**.

*31.3*. When separates from periodicals or other works placed on sale are issued in advance, the date on the separate is accepted as the date of effective publication unless there is evidence that it is erroneous.

Recommendation 31A

*31A.1*. The date on which the publisher or publisher's agent delivers printed matter to one of the usual carriers for distribution to the public should be accepted as its date of effective publication.

## Best practice

Authors of new names, editors and publishers will all be interested in ensuring that the publications including new names are in accordance with the *Melbourne Code*, so that the names therein are effectively published. We suggest that those publishing in journals or monograph series and books that have online editions communicate with the editors so that best practice can be established across the community as quickly as possible. Many publishers have been carefully addressing the issues involved with the e-publication of novelties for some time (see [3]; guidelines in PLoS One [4]) and considerable interest in making these new *Code *changes function effectively has been apparent.

Some practices that we feel will help with the initial stages of e-publication of novelties that are according to the *Melbourne Code *are:

• Having each article bear the date of publication prominently (as is done in many journals, for example *New Phytologist *or *Nature*).

• If an online early version is issued that is not the same as the final version (and thus not the place of effective publication), stamp each article with this fact prominently (for example *American Journal of Botany*).

• Prominent display of the ISSN or ISBN of the publication on each article will help indexers establish effective publication.

• Publication in journals (or monograph series) that participate in the CLOCKSS system (see [3] for a description) or another international archive and preservation system will ensure long-term archiving.

• Authors of new names by electronic means should alert the appropriate indexing center as recommended in Rec. 30A.3 - this will help indexers who may otherwise not be aware of electronically published names.

## What these changes do *not *mean

Although the new Articles and Recommendations use the terms PDF and PDF/A, this does not mean that publications must be issued *only *in that format to be effectively published. For example, some online journals issue papers in Hypertext Markup Language (HTML) format together with a parallel PDF version. In such cases, the PDF version will be effectively published. The stipulation that the General Committee for Botanical Nomenclature will communicate the acceptability of a new international standard format, should PDF ever be succeeded, means authors of novelties and the community using the *Code *can remain informed as to advances in the field and that the *Code *will be protected from obsolescence.

Use of the following means of electronic publication will *not *result in effective publication of novelties under the *Melbourne Code*:

• Publication on websites or in ephemeral documents available over the Internet (there are strict criteria for granting of ISSNs [5]).

• Publication in journals without a registered ISSN or e-ISSN.

• Publication in books without a registered ISBN or e-ISBN.

The Recommendation approved to advise the deposition of a hard copy of any e-publication in a library suggests to botanists an action, but it does not set out standard practice or a protocol for librarians to follow. Librarians are themselves in a complex transition zone between publication modalities [6], and botanists may find librarians to be unwilling or unable to accommodate single hard copy papers as individual accessions should the volume be great.

## Two other important changes to the *Code *relating to the publication of names

The second change to the *Code *approved in Melbourne to take effect from 1 January 2012 is that the description or diagnosis required for valid publication of the name of a new taxon of all organisms falling under the *Code *may be in either English or Latin. This is the current provision for names of plant fossils, but all new non-fossil taxa have required a Latin description or diagnosis (fungi and plants from 1 January 1935; algae [including cyanobacteria, if treated under the *Code*] from 1 January 1958). This has no bearing on the form of scientific names, which continue to be Latin or treated as Latin. Individual journal requirements for Latin and/or English will, of course, be determined by the editors of those journals.

A third change to the *Code *approved in Melbourne relating to publication of names, but one not taking effect until 1 January 2013 (not 1 January 2012 as reported by [7]), is that all new names of organisms treated as fungi must, as an additional requirement for valid publication, include in the protologue (everything associated with a name at its valid publication) the citation of an identifier issued by a recognized repository (such as MycoBank [8]). This will be publicized separately.

The requirement for a unique identifier for new names of fungi on or after 1 January 2013 does *not *apply to plants or algae; there is no need for authors of new names in these groups to request Life Science Identifiers (LSIDs) - or other identifiers - from indexing centers.
